# Scorpion Venom Antimicrobial Peptides Induce Siderophore Biosynthesis and Oxidative Stress Responses in Escherichia coli

**DOI:** 10.1128/mSphere.00267-21

**Published:** 2021-05-12

**Authors:** Mohamed M. Tawfik, Magnus Bertelsen, Mohamed A. Abdel-Rahman, Peter N. Strong, Keith Miller

**Affiliations:** a Biomolecular Sciences Research Centre, Sheffield Hallam University, Sheffield, United Kingdom; b Zoology Department, Faculty of Science, Port Said University, Port Said, Egypt; c Zoology Department, Faculty of Science, Suez Canal University, Ismailia, Egypt; University of Rochester

**Keywords:** AMPs, *E. coli*, scorpion venom, oxidative stress, microarray analysis, RT-PCR, *Escherichia coli*, antimicrobial peptides, microarrays

## Abstract

The increasing development of microbial resistance to classical antimicrobial agents has led to the search for novel antimicrobials. Antimicrobial peptides (AMPs) derived from scorpion and snake venoms offer an attractive source for the development of novel therapeutics. Smp24 (24 amino acids [aa]) and Smp43 (43 aa) are broad-spectrum AMPs that have been identified from the venom gland of the Egyptian scorpion Scorpio maurus *palmatus* and subsequently characterized. Using a DNA microarray approach, we examined the transcriptomic responses of Escherichia coli to subinhibitory concentrations of Smp24 and Smp43 peptides following 5 h of incubation. Seventy-two genes were downregulated by Smp24, and 79 genes were downregulated by Smp43. Of these genes, 14 genes were downregulated in common and were associated with bacterial respiration. Fifty-two genes were specifically upregulated by Smp24. These genes were predominantly related to cation transport, particularly iron transport. Three diverse genes were independently upregulated by Smp43. Strains with knockouts of differentially regulated genes were screened to assess the effect on susceptibility to Smp peptides. Ten mutants in the knockout library had increased levels of resistance to Smp24. These genes were predominantly associated with cation transport and binding. Two mutants increased resistance to Smp43. There was no cross-resistance in mutants resistant to Smp24 or Smp43. Five mutants showed increased susceptibility to Smp24, and seven mutants showed increased susceptibility to Smp43. Of these mutants, formate dehydrogenase knockout (*fdnG*) resulted in increased susceptibility to both peptides. While the electrostatic association between pore-forming AMPs and bacterial membranes followed by integration of the peptide into the membrane is the initial starting point, it is clear that there are numerous subsequent additional intracellular mechanisms that contribute to their overall antimicrobial effect.

**IMPORTANCE** The development of life-threatening resistance of pathogenic bacteria to the antibiotics typically in use in hospitals and the community today has led to an urgent need to discover novel antimicrobial agents with different mechanisms of action. As an ancient host defense mechanism of the innate immune system, antimicrobial peptides (AMPs) are attractive candidates to fill that role. Scorpion venoms have proven to be a rich source of AMPs. Smp24 and Smp43 are new AMPs that have been identified from the venom gland of the Egyptian scorpion *Scorpio maurus palmatus*, and these peptides can kill a wide range of bacterial pathogens. By better understanding how these AMPs affect bacterial cells, we can modify their structure to make better drugs in the future.

## Abstract (precis)

The development of life-threatening resistance of pathogenic bacteria to the antibiotics typically in use in hospitals and the community today has led to an urgent need to discover novel antimicrobial agents with different mechanisms of action. As an ancient host defense mechanism of the innate immune system, antimicrobial peptides (AMPs) are attractive candidates to fill that role.

## INTRODUCTION

The development of life-threatening resistance of pathogenic microorganisms to classic antibiotics has led to an urgent need to discover novel antimicrobial agents with different mechanisms of action (1). As an ancient host defense mechanism of the innate immune system, antimicrobial peptides (AMPs) are attractive candidates to fill that role (2–4). Although AMPs come in many shapes and sizes, the majority are amphipathic alpha-helical proteins with a net positive charge that selectively bind to bacterial cell membranes, resulting in membrane disruption and direct cell lysis. They are reported to do this through a diverse variety of mechanisms, from forming pores to acting as nonspecific detergents. In contrast, a smaller group of nonlytic AMPs are neutral, proline-rich peptides that selectively translocate across bacterial cell membranes, either by receptor-mediated processes or by forming transient, nondisruptive, membrane pores. Once inside the cell, these peptides most often bind to DNA and RNA, resulting in inhibition of nucleotide biosynthesis or subsequent protein misfolding (5). They can also inhibit cell wall and protein biosynthesis.

Scorpion venoms have proven to be a rich source of AMPs, which typically belong to the first group, the amphipathic alpha-helical proteins, interacting with lipid bilayers and destabilizing membranes, resulting in bacterial lysis. Smp24 (24 amino acids [aa]) and Smp43 (43 aa) are novel amphipathic cationic alpha-helical AMPs that have been identified from the venom gland of the Egyptian scorpion Scorpio maurus *palmatus* (6, 7). They have broad-spectrum antimicrobial activity on both Gram-positive and Gram-negative bacteria with MICs ranging from 4 to 128 μg/ml (8). However, in common with most AMPs, potency against Gram-negative pathogens is limited. Both Smp24 and Smp43 have good MIC values (4 to 8 μg/ml) for Smp24 against Bacillus subtilis and Staphylococcus aureus (8) which compare extremely favorably with other scorpion venom AMPs (7). Understanding the mechanism of action of AMPs is crucial in their development as potential antimicrobial agents, and we have previously shown, using atomic force microscopy and quartz crystal microbalance dissipation, that these peptides create pores in synthetic prototypical prokaryotic membranes and induce the formation of nonlamellar lipid structures (8, 9).

Bacteria have evolved mechanisms to recognize and respond to AMPs that manage to penetrate cell membranes by differentially regulating genes in response to generalized membrane stress or as a unique resistance mechanism against specific AMPs (10–12). Here, we have extended our previous model lipid studies to investigate the effects of Smp peptides on native bacteria. Using Escherichia coli as a model for pathogenic Gram-negative bacteria, we have examined the differential expression of E. coli genes on cells exposed to Smp24 and Smp43, using DNA microarray analysis.

Our results suggest that Smp24 and Smp43 not only disrupt bacterial cell membranes but may also interfere with intracellular gene expression, combining mechanistic aspects of both lytic and nonlytic AMPs.

## RESULTS

### Determination of subinhibitory concentrations.

Killing curves were performed to identify subinhibitory concentrations of Smp24, Smp43, and polymyxin B against E. coli. Antimicrobials at subinhibitory concentrations may act as stress inducers or signaling molecules that induce the expression of specific genes that help in understanding the mechanism of action of many antimicrobial agents (13, 14). Subinhibitory concentrations were determined as concentrations that reduced the growth rate of E. coli over a 4- to 5-h time period by half compared with untreated controls. The growth of E. coli was reduced at Smp24, Smp43, and polymyxin B concentrations of 12, 7, and 0.18 μg/ml, respectively.

### Microarray gene expression analysis.

The satisfactory purity and quality of all prepared RNA samples for microarray analysis were confirmed. Quality control reports showed high quality hybridization represented by the level and distribution of the detected signals, and the spike-in linearity plot reflected the accuracy and reproducibility of the probe signals. Real-time PCR (RT-PCR) analysis showed that the levels of expression of the assayed genes in cells treated with Smp peptides were comparable with the microarray analysis results (data not shown).

In total, 313 significantly differentially expressed transcripts were identified, distributed over the three comparisons for each peptide against the respective inhibitor-free controls. Smp24 treatment revealed 130 differentially expressed transcripts (109 coding for known RefSeq genes), Smp43 induced 84 differentially expressed transcripts (68 known RefSeq genes), and polymyxin B induced 99 differentially expressed transcripts (76 known RefSeq genes) (see Fig. S1 to S3 in the supplemental material).

10.1128/mSphere.00267-21.1 FIG S1 Growth of E. coli K-12. Treatment with Smp24 at a concentration of 12 μg/ml (top), Smp43 at 7 μg/ml (middle), and polymyxyin B at 0.18 μg/ml (bottom). Download FIG S1, DOCX file, 0.03 MB. Copyright © 2021 Tawfik et al. 2021 Tawfik et al. https://creativecommons.org/licenses/by/4.0/ This content is distributed under the terms of the Creative Commons Attribution 4.0 International license.

**FIG 1:**
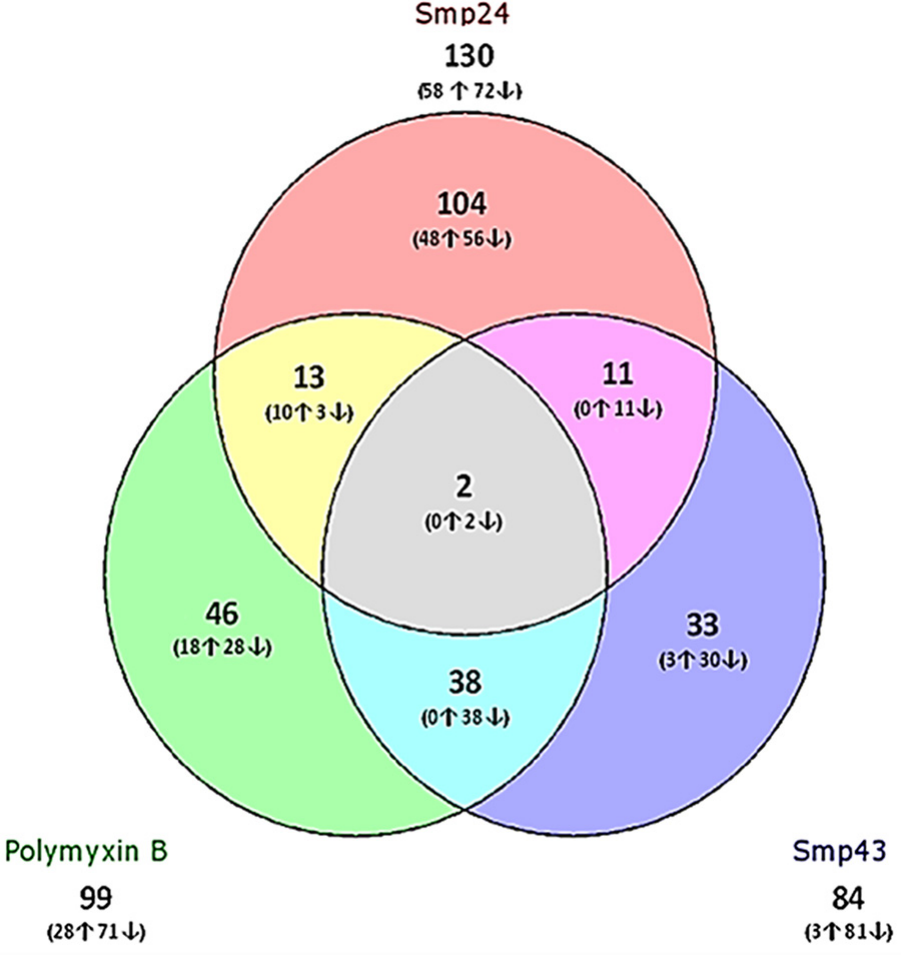
Distribution of significantly differentially expressed transcripts. The three different treatments (Smp24, Smp43, and polymyxin B) were assigned and analyzed compared with untreated E. coli.

Sixty-nine transcripts were upregulated by all three peptides. Forty-eight unique upregulated transcripts were identified for Smp24 treatment, in contrast to only 3 and 18 unique transcripts for Smp43 and polymyxin B, respectively (Fig. 1). Ten upregulated transcripts were induced in common by Smp24 and polymyxin B, whereas no upregulated genes were induced in common by either a combination of Smp43 and polymyxin or a combination of Smp24 and Smp43 (Table 1).

**TABLE 1:** Fold change values of most differentially expressed gene following exposure to Smp24, Smp43, and polymyxin B

Category and gene symbol	Microarray fold change			Gene description
	Smp24	Polymyxin B	Smp43	
Upregulated				
* cirA*	56	39		Ferric iron-catecholate outer membrane transporter
* ECs0636*	20	15		Hypothetical protein
* entA*	18			2,3-Dihydro-2,3-dihydroxybenzoate dehydrogenase
* entB*	19	12		2,3-Dihydro-2,3-dihydroxybenzoate synthase
* entC*	33			Isochorismate synthase
* entE*		11		2,3-Dihydroxybenzoate-AMP ligase
* entF*	17	14		Enterobactin synthetase component F
* feoA*		9		Ferrous iron transporter
* feoC*		8		Orf, hypothetical protein
* fepA*	35	23		Iron-enterobactin outer membrane transporter
* fiu*	39			Predicted iron outer membrane transporter
* flgG*			3	Flagellar biosynthesis
* nrdI*	19			Orf, hypothetical protein
* ubiH*			2	2-Octaprenyl-6-methoxyphenol hydroxylase
* ybdB*	23	18		Hypothetical protein YbdB
* ybiL*	33			Predicted iron outer membrane transporter
* yncE*		10		Putative receptor
Downregulated				
* betB*		127	151	NAD^+^-dependent betaine aldehyde dehydrogenase
* betI*			87	Probably transcriptional repressor of *bet* genes
* betT*		107		High-affinity choline transport
* ccdB*			52	Plasmid maintenance protein
* Crl*		109		Transcriptional regulator of cryptic *csgA* gene
* ECs0346*			31	Putative transporter
* ECs0360*			30	High-affinity choline transport
* ECs0981*	5			Anaerobic dimethyl sulfoxide reductase subunitC
* fdnG*	11			Formate dehydrogenase-N
* fdnH*	7			Formate dehydrogenase-N
* fdnI*	9			Formate dehydrogenase-N
* flmC*		98	88	Hypothetical protein
* frsA*		135	111	Orf, hypothetical protein
* ftnA*	9			Cytoplasmic ferritin
* gpt*		117	109	Guanine-hypoxanthine phosphoribosyltransferase
* intF*			30	Putative phage integrase
* lacI*		226	948	Transcriptional repressor of the Lac operon
* lacZ*			54	Beta-d-galactosidase
* narG*	45		39	Nitrate reductase 1, alpha subunit
* narH*			42	Nitrate reductase 1, beta subunit
* narJ*	27			Nitrate reductase 1, delta subunit
* pepD*		94	372	Aminoacyl-histidine dipeptidase
* proB*			37	Gamma-glutamate kinase
* sopA*		90	100	Plasmid partitioning protein
* sopB*		80		Plasmid partitioning protein
* soxS*	5			Regulation of superoxide response regulon
* tdcF*	24			Orf, hypothetical protein
* yagN*		94	92	Orf, hypothetical protein
* yahK*			62	Putative oxidoreductase
* yahN*			34	Putative cytochrome subunit of dehydrogenase dedehydehdehydrogenase
* yeiT*	8			Putative oxidoreductase
* yjjI*	6			Orf, hypothetical protein
* ykfB*		71	54	Orf, hypothetical protein
* ykgF*			72	Orf, hypothetical protein

With Smp24 treatment, there were 56 unique downregulated transcripts, and 30 and 28 unique transcripts for Smp43 and polymyxin B, respectively. Smp43 and polymyxin B share 38 out of 152 downregulated expressed transcripts (25%); in contrast, only 3 and 11 downregulated transcripts were induced in common between Smp24 and Smp43 and between Smp24 and polymyxin B, respectively.

**FIG 2:**
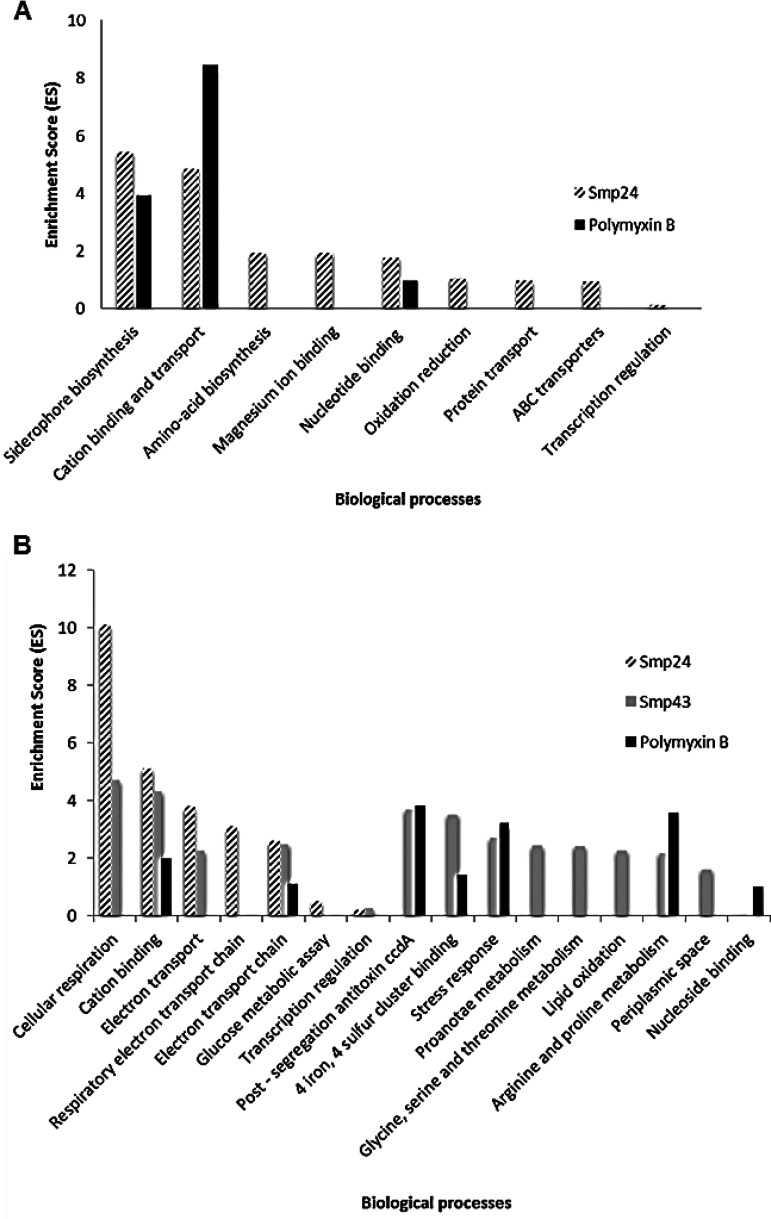
DAVID functional annotation clustering (FAC) analysis of differentially expressed genes obtained by microarray analysis of E. coli following exposures to subinhibitory concentrations of AMPs. (A) Enriched functional gene clusters for the 58 and 28 upregulated genes of Smp24 and polymyxin B, respectively. (B) Enriched functional gene clusters for the 72, 81, and 71 downregulated genes of Smp24, Smp43, and polymyxin B, respectively. Significance is determined by corresponding enrichment scores.

All differentially expressed genes were analyzed using the DAVID analysis tool. Functional annotation clustering (FAC) analysis of unique upregulated genes in response to Smp24 treatment resulted in nine enriched functional clusters under medium stringency. Siderophore biosynthetic processing and di-trivalent ion binding and transport were the most biologically important gene groups with enrichment scores (ES) of 5.45 and 4.86, respectively (*P* < 0.05) for Smp24 (Fig. 2A). FAC revealed four genes in a cluster of siderophore biosynthesis processing and 13 genes in a cluster of cation binding and transport functions. The remaining clusters of upregulated genes were dominated by genes of amino acid biosynthesis, magnesium ion binding, and nucleotide binding. The majority (60%) of enriched clusters identified for polymyxin B upregulated genes were similar to those clustered for Smp24 upregulated genes, and both treatments share clusters related to cation binding, siderophore biosynthesis, and nucleotide binding (Fig. 2A). FAC analysis clustered the common upregulated genes in response to Smp24 and polymyxin B treatments in one enriched functional cluster, including mainly cation binding and transport processes under the medium stringency option (ES = 3.82, *P* < 0.05). Due to the low number of differentially regulated genes following Smp43 exposure, FAC clustering was not possible.

FAC analysis of downregulated genes of Smp24 generated seven enriched clusters. The highest enriched gene group was cellular respiration (ES = 10.11), followed by cation (iron) binding and transport (ES = 5.1). Some other clusters with lower ES were mainly related to cellular respiration such as electron transport and formate dehydrogenase (NAD^+^) activity. All of these clusters are shown in Fig. 2B. Smp43 downregulated genes were clustered into 14 enriched clusters, the highest enrichment scores were for clusters relating to cellular respiration and cation (iron) binding, the same as identified for Smp24. The next highest Smp43 downregulated gene groups according to ES values were postsegregation antitoxin *ccdA*, stress response, electron transport chain pathways. Clusters such as postsegregation antitoxin *ccdA* (ES = 3.84), arginine and proline metabolism (ES = 3.59), stress response (ES = 3.21), cation binding (ES = 1.99), iron-sulfur cluster binding (ES = 1.4), and electron transport chain (ES = 1.1) were also identified for polymyxin B downregulated genes (Fig. 2B).

Interestingly, the biological processes of cation binding, cellular respiration, and electron transport were the most affected processes, either up- or downregulated in response to both Smp24 and Smp43 treatments.

### Screening the Keio collection.

In order to identify the phenotypic effect of genes predicted to be differentially regulated by Smp peptide treatment, the effects of Smp peptides on knockout strains from the Keio E. coli collection, selected on the basis of differential regulation of these genes following exposure to the peptides, were examined. The sensitivity profile of Smp24 was determined against 47 single-gene knockouts. Only 15 mutants showed different MICs against Smp24 compared with the wild-type parent Keio strain (MG1655, MIC = 32 μg/ml). Ten mutant strains were more resistant to Smp24 and had an increased MIC (64 μg/ml) with respect to the parent strain. The majority (>75%) of these genes (*fiu*, *entC*, *entA*, *entB*, *entH*, *fepA*, and *fhuA*) are involved in the biosynthesis of siderophores and cation transport. Additionally, the deletion of the protein binding gene *fimC* increased resistance to Smp24, as did the deletion of *tatE*, a component of TatABCE protein export complex (Table 2).

**TABLE 2:** Susceptibility profile of Keio mutant strains to Smp peptides

Susceptibility	Peptide	Functional group	Gene function	Gene knockout	Fold change vs MIC
Increased susceptibility	Smp24	Oxidative stress response	Global transcription regulator for superoxide response	*soxS*	≤0.5×
			Superoxide dismutase; response to oxidative stress	*sodB*	≤0.5×
		Anaerobic respiration	Formate dehydrogenase-N	*fdnG*	≤0.5×
			Heme exporter subunit;cytochrome *c* biogenesis system	*ccmB*	≤0.5×
		Unknown function	Unknown function	*ycgK*	≤0.5×
	Smp43	Oxidative stress response	2-Octaprenyl-6-methoxyphenol hydroxylase	*ubiH*	≤0.5×
		Anaerobic respiration	Dimethyl sulfoxide reductase	*dmsA*	≤0.5×
			Formate dehydrogenase-N	*fdnH*	≤0.5×
			Metal ion binding	*hypA*	≤0.5×
			Quinol dehydrogenase, electron source for NapAB; cytochrome *c*-type protein (electron carrier)	*napC*	≤0.5×
			Formate dehydrogenase-N	*fdnG*	≤0.5×
			Glycerate kinase	*garK*	≤0.5×
Increased resistance	Smp24	Siderophore transport and biosynthesis	Putative outer membrane receptor for iron transport	*Fiu*	≥2×
			Outer membrane receptor for ferric enterobactin	*fepA*	≥2×
			Outer membrane ferrichrometransport system	*fhuA*	≥2×
			Isochorismate synthase	*entC*	≥2×
			2,3-Dihydro-2,3-dihydroxybenzoate dehydrogenase	*entA*	≥2×
			2,3-Dihydro-2,3-dihydroxybenzoate synthase; asochorismatase	*entB*	≥2×
			Thioesterase required for efficient enterobactin production	*ybdB* (*entH*)	≥2×
		Protein transporter activity	Component of TatABCE protein export complex; Protein translocase	*tatE*	≥2×
	Smp43	Nuclease activity	Putative DNase (nuclease activity)	*yjjV*	≥2×
		Protein binding	Periplasmic chaperone	*fimC*	≥2×
		Unknown function	Unknown function	*ykfB*	≥2×
		Magnesium ion binding	Glutamate 5-kinase, proline biosynthesis	*proB*	≥2×

Knocking out genes *sodB*, *fdnG*, *ycgK*, *soxS*, and *ccmB* resulted in cellular sensitivity to Smp24 with a twofold decrease in the MIC compared with the wild-type strain. Two of these genes (*sodB* and *soxS*) play an important role in bacterial protection from superoxide radicals; however, *fdnG* and *ccmB* are involved in the nitrate reduction process during anaerobic respiration (Table 2). Forty-one strains from the Keio knockout strains were tested for Smp43 susceptibility. Only nine mutants displayed different MICs against Smp43. The single deletion mutants of the genes *napC*, *garK*, *fdnG*, *dsmA*, *hypA*, and *fdnH*, which are involved in anaerobic respiration, and oxidoreductase gene *ubiH*, implicated in response to oxidative stress, exhibited increases in susceptibility to Smp43 (16 μg/ml). Two strains showed increased resistance, one of these carried a deletion of *proB*, a gene that has a function in magnesium ion binding and kinase activity. The other resistant strain has a deletion of an uncharacterized “hypothetical” gene, *ykfB* (Table 2). To ensure that any changes in susceptibility to Smp peptides of the knockout strains selected from the Keio collection were not a consequence of the genetic modification process, the growth rates of all strains were compared with the growth rate of the unmodified parental strain. Cross-resistance studies were conducted using ampicillin, ciprofloxacin, and doxycycline (three antibiotics with unrelated mechanisms of action compared with each other or with the Smp peptides), and all of the constructs had the same growth rate and susceptibility profile as the parent strain (data not shown).

## DISCUSSION

Antimicrobial agents may act as stress inducers at subinhibitory concentrations, and microarray analysis has identified many bacterial genes that are either up- or downregulated by a variety of antibiotics, helping in turn to enable a detailed understanding of their mechanism of action (13–16). Conventional antibiotics work by directly targeting a specific target within the bacterial cell. Pore-forming antimicrobial peptides are a promising new avenue for the development of novel antimicrobials because the nonspecific pores that they create in the bacterial cell membrane allow intracellular contents to leak out, resulting in the generalized metabolic death of the bacterium. Although many studies have examined pore formation, it is unclear whether this, rather nonspecific event, is the sole contributor to bacterial cell death, or whether AMPs themselves, either directly or indirectly, alter gene expression, and consequently cell metabolism, before catastrophic membrane disruption occurs—and thereby possibly contributing to the final destruction of the cell from an intracellular perspective.

Scorpion venoms are a rich source of pore-forming AMPs, and we have extensively studied peptide-membrane interactions of two of these (Smp24 and Smp43) peptides from the Egyptian scorpion *Scorpio maurus palmatus*. Here, we have sought to examine whether sublethal exposure to Smp24 and Smp43 affects transcriptomic responses of E. coli using microarray analysis as a rapid and cost-effective method to evaluate global expression profiles and thus provide evidence for a potential additional, secondary mechanism of action for pore-forming AMPs. In our analysis, we have compared our scorpion venom AMPs with polymyxin B which, although structurally unrelated, is one of a small number of pore-forming antimicrobials in therapeutic use. By including polymyxin B, we sought to eliminate generalized transcriptomic responses to peptide-induced membrane damage to allow for a clearer analysis of Smp peptide-specific responses that may shed light on secondary mechanisms of action.

Sublethal doses of Smp24, Smp43, and polymyxin B all alter E. coli gene transcription, although each by a different mechanism. A common tenet of biology today is that the function of a peptide or protein correlates with its structure, an idea developed from the recognition of the importance of gene structure in being able to predict protein function. However, it must be realized that the holistic term antimicrobial peptide has been applied to characterize an incredibly diverse set of genes that are related only because they encode molecules that can kill or inhibit the growth of microbes. Cationic scorpion venom AMPs are typical in this respect, and biological activity depends on the three-dimensional alignment of hydrophobic and basic amino acid residues on opposing faces of the peptide. Consequently, the transcriptomic responses of Smp24 and Smp43 are quite different, even though they both adopt amphipathic alpha-helical structures in the nonpolar environment of a membrane.

Most bacterial genes differentially expressed in response to treatment with Smp peptides were stress genes that have previously been upregulated during siderophore biosynthesis (17, 18), as well as cation binding and transport. Genes implicated in antioxidant responses and anaerobic respiration were also prominent. Our findings suggest that an imbalance in iron homeostasis coupled with oxidative stress may be involved in E. coli responses to Smp peptides. In order to increase iron uptake, bacteria synthesize siderophores to chelate Fe^3+^ iron and make complexes (19). The Ent gene cluster encodes proteins related to the biosynthesis of enterobactin (20–22), and in the present study, *entC*, *entA*, and *entH* were uniquely upregulated in response to Smp24 stress. This suggests that Smp24 stimulates the production of siderophores in *E.coli* and that siderophore transport mechanisms may have a role in the antimicrobial activity of this peptide.

Fe^3+^-siderophore complexes cross the Gram-negative outer membrane through specific membrane receptors and are translocated to the cytoplasm by inner membrane transporter proteins in order to deliver iron (19, 23). Four genes encoding siderophore receptors (*fiu*, *fepA*, *cirA*, and *fhuA*), as well as the siderophore transporter, *fepC*, were significantly overexpressed in response to Smp24, compared with untreated cells. Siderophore biosynthesis is regulated by the Fe^3+^ uptake regulator (Fur) system, as Fur cluster genes repress siderophore biosynthesis according to the availability of iron (24, 25). Fur is regulated by the oxidative stress response regulon *soxS* (26), as well as having roles in cellular oxidative stress defense such as the activation of superoxide dismutase *sodB* (27, 28). Both *soxS* and *sodB* were significantly downregulated in response to Smp24 treatment, and the downregulation of both these oxidative stress response genes leads directly to lower bacterial resistance to oxidative stress. Similarly, the deletion of oxidative response gene *ubiH* generated a higher susceptibility to Smp43.

Functional annotation clustering (FAC) analysis showed that the Fe-S cluster was significantly downregulated in response to both Smp peptide treatments, again supporting the involvement of oxidative stress. The sensory domain of fumarate and nitrate reductase (FNR), which is a transcriptional regulator for anaerobic growth, contains an Fe-S cluster, and Fe-S inactivation inhibits the switch from aerobic growth (29). Under aerobic conditions, bacteria rapidly acquire and oxidize iron in a siderophore-mediated process (30). During anaerobic conditions, Fe^2+^ levels are stable and in soluble form (31, 32). The process of Fe^3+^ solubilization during anaerobic respiration is not siderophore dependent (33). Gene clusters, mainly related to the electron transport chain and cellular respiration, were also found to be abundant in the analysis of downregulated genes in response to Smp24 and Smp43, which may explain the highly significant downregulation of anaerobic-related genes following exposure to sublethal doses of both peptides.

The results of experiments with the Keio collection of E. coli mutants support these hypotheses. Knockout mutants of genes from the enterobactin gene cluster (*ΔentB*, *ΔentH*, *ΔentC*, and *ΔentA*) and iron-regulated outer membrane receptors (*ΔfepA*, *Δfiu*, and *ΔfhuA*) showed a marginally higher, but nevertheless significant, level of resistance to Smp24. Deletion of either the *sodB* superoxide dismutase gene or the *soxS* oxidative stress response regulon gene resulted in increased cellular sensitivity to Smp24 compared with the wild-type strain, and similarly, deletion of the *ubiH* oxidative response gene generated higher susceptibility to Smp43. In general, all genes related to oxidative stress responses showed a marginally higher level of sensitivity to either Smp24 or Smp43 compared with the parent strain. Mutants with deletions related to anaerobic respiration also resulted in a decreased MIC for either peptide.

It is interesting to note that polymyxin B, a structurally unrelated pore-forming lipopeptide, also induced downregulation of siderophore genes *entB* and *entF*, as well as upregulating the siderophore membrane transporters *fepA* and *cirA*, in common with Smp24. In common with both Smp24 and Smp43, gene clusters related to the electron transport chain (e.g., *porfE*) and cellular respiration were also found to be abundant in the analysis of downregulated genes in response to polymyxin (34). The antimicrobial activity of a modified form of microcin E492, another pore-forming AMP, is also mediated by FepA, Cir, and Fiu (35–38). Microcins are rich in serine and glycine residues with a conserved serine-rich C terminus (39). Both Smp24 and Smp43 have serine residues in their C-terminal tail and are rich in serine and glycine amino acids that may enhance binding to siderophores to form Smp24-siderophore conjugates that facilitate their delivery intracellularly. The siderophore outer membrane receptor FhuA is also the target of the AMP microcin J25, which induces oxidative stress through the increased production of reactive oxygen species (ROS) (40, 41). AMPs exert their effects through multiple complex modes of action. The activities of AMPs are typically reported as being dependent upon an initial interaction with the bacterial cell membrane (42). However, the upregulation of siderophore biosynthesis in the presence of Smp peptides and the reduction in the susceptibility of strains deficient in siderophore transport genes support the proposal that these peptides are transported, either independently or in complex with siderophores, via iron transport mechanisms. Additionally, the upregulation of cation transport and oxidative stress response genes, coupled with the increased sensitivity of E. coli strains with deletions of these upregulated genes to both Smp peptides, is consistent with the observation that a major component of AMP activity is linked to ROS generation. While the electrostatic association between pore-forming AMPs and bacterial membranes followed by integration of the peptide into the membrane is the initial starting point, it is clear that there are numerous subsequent additional intracellular mechanisms that contribute to their overall antimicrobial effect.

One of the biggest drawbacks for proposing native AMPs as broad-spectrum antibiotics has been their limited potency against Gram-negative bacterial pathogens. Poor stability of AMPs in serum has also limited their use as novel therapeutics. However, disulfide-bridged peptides are more resistant to protease degradation than linear peptides; therefore, joining the terminal ends of peptides by disulfide bridges (cyclization) constrains the peptide structure and thereby enhances antimicrobial activity (43, 44). The delivery of AMPs is another crucial factor limiting their clinical application. Systemic and presystemic enzymatic degradation and rapid hepatic and renal removal from the circulation are challenging for oral and systemic administration routes. Therefore, the most common administration route of AMPs is localized topical application (45).

Smp43 has a helix-hinge-helix topology, characteristic of several longer-chain AMPs, all of which have respectable therapeutic indices, for example, pandinin-1 (46) and dermaseptin B2 (47). In contrast to Smp43, Smp24 is a single alpha-helical peptide, and it has been demonstrated that the potency of Smp24 can be increased by increasing the positive charge density at its N terminus (48). Increasing the hydrophobicity of Smp24 reduced its cytotoxicity to a level comparable to those of established pore-forming antibiotics such as daptomycin and polymyxin B (48). These studies suggest that, used as templates, this class of AMPs has considerable potential for the development of effective anti-infective agents.

Although more than 3,000 AMPs have been discovered thus far, there are several challenges for the large-scale development of antimicrobial peptides for therapeutic applications, and these have been discussed recently (49). Those AMPs in clinical use are primarily restricted to topical applications because, as peptides, most AMPs are susceptible to nonspecific degradation by intracellular proteases. It has been recognized that venom peptides with multiple disulfide bonds have remarkable thermal stability and resistance to proteases; as a result, several disulfide-bridged venom peptides are in use in a variety of clinical applications (e.g., ziconotide for severe chronic pain and eptifibatide for acute coronary syndrome) (50). The cyclization of linear AMPs has been shown both to increase peptide stability and to increase antimicrobial activity (44, 51, 52); it will be interesting to see whether chemical modification of Smp24 and Smp43 to produce disulfide-bridged or cyclical peptides will increase antimicrobial activity as well as improve resistance to proteases.

## MATERIALS AND METHODS

### Peptide synthesis.

Peptides were synthesized by solid-phase 9-fluorenylmethoxy carbonyl (FMOC) synthesis (ProImmune Limited, Oxford, UK) with >90% purity. The Smp24 peptide (IWSFLIKAATKLLPSLFGGGKKDS) and the Smp43 peptide (GVWDWIKKTAGKIWNSEPVKALKSQALNAAKNFVAEKIGATPS) were used.

### Bacterial growth conditions and determination of subinhibitory concentrations.

Four independent cultures of the E. coli strain JM109 were exposed to various subinhibitory concentrations of each Smp peptide, and polymyxin B as positive control, in order to identify subinhibitory concentrations of peptides that lowered growth rates to 50%. A culture of E. coli JM109 was grown overnight in Mueller-Hinton broth and then diluted into fresh broth (2 × 10^7^ CFU/ml). The culture was then exposed to each peptide, incubated at 37°C with shaking, and grown to the exponential growth phase (*A*_600_ = 0.6). Untreated bacteria were used as negative controls.

### Gene expression microarray analysis.

A gene expression analysis protocol was followed using a one-color microarray technique (v6.5) (Agilent, Wokingham, UK). Bacterial samples were collected during the mid-logarithmic growth phase. Total RNA was extracted by using an SV total RNA isolation system (Promega Corporation, Madison, WI, USA). Extracted RNA was assessed for concentration, purity, and integrity using an Agilent 2100 bioanalyzer (Agilent, Wokingham, UK) to reduce biases in microarray analysis caused by poor RNA quality. Sample RNA, together with the internal control transcripts, were labeled with cyanine 3-CTP (Cy3) dye during an amplification reaction using an Agilent Low Input Quick Amp kit. The amplified fluorescent cRNA products were then purified (RNeasy minikit; Qiagen), and the Cy3 specific activity was quantified by absorbance at 260 nm. Each slide was hybridized with 600 ng Cy3-labeled cRNA for 17 h (Gene Expression Hybridization kit; Agilent). Hybridized slides were disassembled, washed, and then scanned at 3-μm resolution (Agilent C microarray scanner), preset with the default settings for an 8 × 15,000 (15K) microarray format. All samples were tested in duplicate on each of two separate arrays.

### Data analysis.

Agilent feature extraction (FE) software (version 10.7) was used to process the generated images. Raw “txt” data files were analyzed (Agilent GeneSpring GX software, version 13.1; Agilent, Santa Clara, CA). Briefly, intensity values were subjected to a log_2_ transformation and normalized to the 75th percentile-shift normalization. The imported data files were then grouped and assigned to the experiment parameters and conditions. Differentially expressed genes were statistically selected with *P* < 0.01 by using unpaired *t* test and applying a Benjamini-Hochberg multiple testing correction method to correct the computed *P* values. Finally, the list of the identified genes was filtered to include only genes with a ≥2-fold change.

### Gene cluster enrichment analysis.

The DAVID database of software tools was used for gene and molecular pathway analysis (https://david.ncifcrf.gov/home.jsp). Enrichment analyses of the differentially expressed genes were performed using DAVID 6.7 with the thresholds of *P* value of <0.05 and enrichment gene count of >2. Functional annotation clustering (FAC) allowed clustering of Gene Ontology (GO) categories sharing a significant number of genes.

### Validation of microarray data by real-time PCR (RT-PCR).

The expression levels of four representative genes (two upregulated genes, *fiu* and *fepA*, and two downregulated genes, *proB* and *fdnG*) were examined by RT-PCR to validate the microarray expression data. RNA was reverse transcribed into cDNA with a QuantiTect reverse transcription kit (Qiagen Inc., Chatsworth, CA, USA). RT-PCRs were performed on a StepOnePlus real-time PCR system, using TaqMan probes (Applied Biosystems, CA, USA). *cysG*, *idnT*, and *hcat* were used as housekeeping genes (53). Results were analyzed using the 2^–ΔΔCt^ method and presented as relative gene expression normalized to the average cycle threshold for the three housekeeping genes. To test the primers for specificity and quality, efficiency curves were performed, and samples were run in triplicate.

### Keio collection screen.

In order to identify essential genes whose products were involved in the response of E. coli when treated by Smp peptides, the susceptibility of 88 E. coli single-knockout mutant strains from the Keio collection (National BioResource Project, National Institute of Genetics, Japan) was assayed using the BSAC broth microdilution technique (54). The preselection of the mutants for the assay was based on the differentially expressed genes identified from microarray analysis. Strains with knockouts in the identified genes were compared with that of the Keio collection E. coli MG1655 parent strain. Eight Keio strains with genes not identified as significant in the microarray screen (*ihfB*, *uxuR*, *hacT*, *cysG*, *ugpQ*, *idnT*, *yghB*, and *pbpC*), were selected as controls. The MIC was determined as the lowest concentration of peptides for which no growth was observed.

10.1128/mSphere.00267-21.2 FIG S2 RT-PCR analysis of the relative mRNA expression levels of selected upregulated genes in polymyxin B-, Smp24-, and Smp43-treated E. coli compared with an untreated control. (A) Relative expression of *fepA* mRNA among the different treatments. The inset shows the expression pattern of the gene by microarray. (B) Relative expression of *fiu* among different treatments. The inset shows the expression pattern of the gene by microarray. Data are expressed as the means ± standard errors (SE). Statistical analysis was performed by the Kruskal-Wallis test. Statistical significance is indicated as follows: *, *P* < 0.05; **, *P* < 0.01; ***, *P* < 0.001. Download FIG S2, DOCX file, 0.1 MB. Copyright © 2021 Tawfik et al. 2021 Tawfik et al. https://creativecommons.org/licenses/by/4.0/ This content is distributed under the terms of the Creative Commons Attribution 4.0 International license.

10.1128/mSphere.00267-21.3 FIG S3 RT-PCR analysis of the relative mRNA expression levels of selected downregulated genes in polymyxin B-, Smp24-, and Smp43-treated E. coli compared with an untreated control. (A) Relative mRNA expression of *fdnG* among the different treatments. The inset shows the expression pattern of the gene by microarray. (B) Relative expression of *proB* among different treatments. The inset shows the expression pattern of the gene by microarray. Data are expressed as means ± SE. Statistical analysis was performed by the Kruskal-Wallis test. Statistical significance is indicated as follows: *, *P* < 0.05 compared to untreated control cells; **, *P* < 0.01; ***, *P* < 0.001. Download FIG S3, DOCX file, 0.1 MB. Copyright © 2021 Tawfik et al. 2021 Tawfik et al. https://creativecommons.org/licenses/by/4.0/ This content is distributed under the terms of the Creative Commons Attribution 4.0 International license.

## References

[1] Jodoin J, Hincke MT. 2018. Histone H5 is a potent antimicrobial agent and a template for novel antimicrobial peptides. Sci Rep 8:2411. doi:10.1038/s41598-018-20912-1. 29402952 PMC5799255

[2] Zasloff M. 2002. Antimicrobial peptides of multicellular organisms. Nature 415:389–395. doi:10.1038/415389a. 11807545

[3] Lai Y, Gallo RL. 2009. AMPed up immunity: how antimicrobial peptides have multiple roles in immune defense. Trends Immunol 30:131–141. doi:10.1016/j.it.2008.12.003. 19217824 PMC2765035

[4] Guilhelmelli F, Vilela N, Albuquerque P, da S Derengowski L, Silva-Pereira I, Kyaw CM. 2013. Antibiotic development challenges: the various mechanisms of action of antimicrobial peptides and of bacterial resistance. Front Microbiol 4:353. doi:10.3389/fmicb.2013.00353. 24367355 PMC3856679

[5] Cardoso MH, Meneguetti BT, Costa BO, Buccini DF, Oshiro KG, Preza SL, Carvalho CM, Migliolo L, Franco OL. 2019. Non-lytic antibacterial peptides that translocate through bacterial membranes to act on intracellular targets. Int J Mol Sci 20:4877. doi:10.3390/ijms20194877. PMC6801614 31581426

[6] Abdel-Rahman MA, Quintero-Hernandez V, Possani LD. 2013. Venom proteomic and venomous glands transcriptomic analysis of the Egyptian scorpion Scorpio maurus palmatus (Arachnida: Scorpionidae). Toxicon 74:193–207. doi:10.1016/j.toxicon.2013.08.064. 23998939

[7] Harrison PL, Abdel-Rahman MA, Strong PN, Tawfik MM, Miller K. 2016. Characterisation of three alpha-helical antimicrobial peptides from the venom of Scorpio maurus palmatus. Toxicon 117:30–36. doi:10.1016/j.toxicon.2016.03.014. 27019370

[8] Harrison PL, Heath GR, Johnson BRG, Abdel-Rahman MA, Strong PN, Evans SD, Miller K. 2016. Phospholipid dependent mechanism of smp24, an α-helical antimicrobial peptide from scorpion venom. Biochim Biophy Acta 1858:2737–2744. doi:10.1016/j.bbamem.2016.07.018. 27480803

[9] Heath GR, Harrison PL, Strong PN, Evans SD, Miller K. 2018. Visualization of diffusion limited antimicrobial peptide attack on supported lipid membranes. Soft Matter 14:6146–6154. doi:10.1039/C8SM00707A. 29999090

[10] Brazas MD, Hancock REW. 2005. Using microarray gene signatures to elucidate mechanisms of antibiotic action and resistance. Drug Discov Today 10:1245–1252. doi:10.1016/S1359-6446(05)03566-X. 16213417

[11] Fehri LF, Sirand-Pugnet P, Gourgues G, Jan G, Wroblewski H, Blanchard A. 2005. Resistance to antimicrobial peptides and stress response in Mycoplasma pulmonis. Antimicrob Agents Chemother 49:4154–4165. doi:10.1128/AAC.49.10.4154-4165.2005. 16189093 PMC1251518

[12] Pietiäinen M, Gardemeister M, Mecklin M, Leskelä S, Sarvas M, Kontinen VP. 2005. Cationic antimicrobial peptides elicit a complex stress response in Bacillus subtilis that involves ECF-type sigma factors and two-component signal transduction systems. Microbiology (Reading) 151:1577–1592. doi:10.1099/mic.0.27761-0. 15870467

[13] Fajardo A, Martínez JL. 2008. Antibiotics as signals that trigger specific bacterial responses. Curr Opin Microbiol 11:161–167. doi:10.1016/j.mib.2008.02.006. 18373943

[14] Xu G-M. 2016. Relationships between the regulatory systems of quorum sensing and multidrug resistance. Front Microbiol 7:958. doi:10.3389/fmicb.2016.00958. 27379084 PMC4909744

[15] Linares JF, Gustafsson I, Baquero F, Martinez J. 2006. Antibiotics as intermicrobial signaling agents instead of weapons. Proc Natl Acad Sci U S A 103:19484–19489. doi:10.1073/pnas.0608949103. 17148599 PMC1682013

[16] Bernier SP, Surette MG. 2013. Concentration-dependent activity of antibiotics in natural environments. Front Microbiol 4:20. doi:10.3389/fmicb.2013.00020. 23422936 PMC3574975

[17] Chen L-H, Yang SL, Chung K-R. 2014. Resistance to oxidative stress via regulating siderophore-mediated iron acquisition by the citrus fungal pathogen Alternaria alternata. Microbiology (Reading) 160:970–979. doi:10.1099/mic.0.076182-0. 24586035

[18] Ruiz JA, Bernar EM, Jung K. 2015. Production of siderophores increases resistance to fusaric acid in Pseudomonas protegens Pf-5. PLoS One 10:e0117040. doi:10.1371/journal.pone.0117040. 25569682 PMC4287623

[19] Vassiliadis G, Peduzzi J, Zirah S, Thomas X, Rebuffat S, Destoumieux-Garzón D. 2007. Insight into siderophore-carrying peptide biosynthesis: enterobactin is a precursor for microcin E492 posttranslational modification. Antimicrob Agents Chemother 51:3546–3553. doi:10.1128/AAC.00261-07. 17646411 PMC2043276

[20] Fischbach MA, Lin H, Liu DR, Walsh CT. 2006. How pathogenic bacteria evade mammalian sabotage in the battle for iron. Nat Chem Biol 2:132–138. doi:10.1038/nchembio771. 16485005

[21] Miethke M, Marahiel MA. 2007. Siderophore-based iron acquisition and pathogen control. Microbiol Mol Biol Rev 71:413–451. doi:10.1128/MMBR.00012-07. 17804665 PMC2168645

[22] Salvail H, Lanthier-Bourbonnais P, Sobota JM, Caza M, Benjamin J-AM, Mendieta MES, Lépine F, Dozois CM, Imlay J, Massé E. 2010. A small RNA promotes siderophore production through transcriptional and metabolic remodeling. Proc Natl Acad Sci U S A 107:15223–15228. doi:10.1073/pnas.1007805107. 20696910 PMC2930555

[23] Stintzi A, Barnes C, Xu J, Raymond KN. 2000. Microbial iron transport via a siderophore shuttle: a membrane ion transport paradigm. Proc Natl Acad Sci U S A 97:10691–10696. doi:10.1073/pnas.200318797. 10995480 PMC27084

[24] Crosa JH, Walsh CT. 2002. Genetics and assembly line enzymology of siderophore biosynthesis in bacteria. Microbiol Mol Biol Rev 66:223–249. doi:10.1128/MMBR.66.2.223-249.2002. 12040125 PMC120789

[25] Dale SE, Doherty-Kirby A, Lajoie G, Heinrichs DE. 2004. Role of siderophore biosynthesis in virulence of Staphylococcus aureus: identification and characterization of genes involved in production of a siderophore. Infect Immun 72:29–37. doi:10.1128/IAI.72.1.29-37.2004. 14688077 PMC343950

[26] Pomposiello PJ, Bennik MH, Demple B. 2001. Genome-wide transcriptional profiling of the Escherichia coli responses to superoxide stress and sodium salicylate. J Bacteriol 183:3890–3902. doi:10.1128/JB.183.13.3890-3902.2001. 11395452 PMC95271

[27] da Silva Neto JF, Braz VS, Italiani VC, Marques MV. 2009. Fur controls iron homeostasis and oxidative stress defense in the oligotrophic alpha-proteobacterium Caulobacter crescentus. Nucleic Acids Res 37:4812–4825. doi:10.1093/nar/gkp509. 19520766 PMC2724300

[28] Zhang T, Ding Y, Li T, Wan Y, Li W, Chen H, Zhou R. 2012. A Fur-like protein PerR regulates two oxidative stress response related operons dpr and metQIN in Streptococcus suis. BMC Microbiol 12:85. doi:10.1186/1471-2180-12-85. 22646062 PMC3458967

[29] Unden G, Schirawski J. 1997. The oxygen‐responsive transcriptional regulator FNR of Escherichia coli: the search for signals and reactions. Mol Microbiol 25:205–210. doi:10.1046/j.1365-2958.1997.4731841.x. 9282732

[30] Kouzuma A, Hashimoto K, Watanabe K. 2012. Roles of siderophore in manganese-oxide reduction by Shewanella oneidensis MR-1. FEMS Microbiol Lett 326:91–98. doi:10.1111/j.1574-6968.2011.02444.x. 22092340

[31] Kershaw S, Cundy A. 2000. Oceanography: an earth science perspective. Routledge, Milton, United Kingdom.

[32] Valentine RC, Valentine DL. 2009. Omega-3 fatty acids and the DHA principle. CRC Press, Boca Raton, FL.

[33] Fennessey CM, Jones ME, Taillefert M, DiChristina TJ. 2010. Siderophores are not involved in Fe(III) solubilization during anaerobic Fe(III) respiration by Shewanella oneidensis MR-1. Appl Environ Microbiol 76:2425–2432. doi:10.1128/AEM.03066-09. 20190086 PMC2849222

[34] Sampson TR, Liu X, Schroeder MR, Kraft CS, Burd EM, Weiss DS. 2012. Rapid killing of Acinetobacter baumannii by polymyxins is mediated by a hydroxyl radical death pathway. Antimicrob Agents Chemother 56:5642–5649. doi:10.1128/AAC.00756-12. 22908157 PMC3486575

[35] Thomas X, Destoumieux-Garzon D, Peduzzi J, Afonso C, Blond A, Birlirakis N, Goulard C, Dubost L, Thai R, Tabet JC, Rebuffat S. 2004. Siderophore peptide, a new type of post-translationally modified antibacterial peptide with potent activity. J Biol Chem 279:28233–28242. doi:10.1074/jbc.M400228200. 15102848

[36] Destoumieux-Garzón D, Peduzzi J, Thomas X, Djediat C, Rebuffat S. 2006. Parasitism of iron-siderophore receptors of Escherichia coli by the siderophore-peptide microcin E492m and its unmodified counterpart. Biometals 19:181–191. doi:10.1007/s10534-005-4452-9. 16718603

[37] Nolan EM, Fischbach MA, Koglin A, Walsh CT. 2007. Biosynthetic tailoring of microcin E492m: post-translational modification affords an antibacterial siderophore-peptide conjugate. J Am Chem Soc 129:14336–14347. doi:10.1021/ja074650f. 17973380 PMC2522288

[38] Raines DJ, Moroz OV, Blagova EV, Turkenburg JP, Wilson KS, Duhme-Klair AK. 2016. Bacteria in an intense competition for iron: key component of the Campylobacter jejuni iron uptake system scavenges enterobactin hydrolysis product. Proc Natl Acad Sci U S A 113:5850–5855. doi:10.1073/pnas.1520829113. 27162326 PMC4889360

[39] Vassiliadis G, Destoumieux-Garzón D, Lombard C, Rebuffat S, Peduzzi J. 2010. Isolation and characterization of two members of the siderophore-microcin family, microcins M and H47. Antimicrob Agents Chemother 54:288–297. doi:10.1128/AAC.00744-09. 19884380 PMC2798501

[40] Destoumieux-Garzon D, Duquesne S, Peduzzi J, Goulard C, Desmadril M, Letellier L, Rebuffat S, Boulanger P. 2005. The iron-siderophore transporter FhuA is the receptor for the antimicrobial peptide microcin J25: role of the microcin Val11-Pro16 beta-hairpin region in the recognition mechanism. Biochem J 389:869–876. doi:10.1042/BJ20042107. 15862112 PMC1180738

[41] Mathavan I, Zirah S, Mehmood S, Choudhury HG, Goulard C, Li Y, Robinson CV, Rebuffat S, Beis K. 2014. Structural basis for hijacking siderophore receptors by antimicrobial lasso peptides. Nat Chem Biol 10:340–342. doi:10.1038/nchembio.1499. 24705590 PMC3992131

[42] Jenssen H, Hamill P, Hancock RE. 2006. Peptide antimicrobial agents. Clin Microbiol Rev 19:491–511. doi:10.1128/CMR.00056-05. 16847082 PMC1539102

[43] Brewer D, Lajoie G. 2002. Complexation analysis of the antimicrobial salivary histatin peptides, p 744–745. *In* Peptides for the new millennium. Springer, Dordrecht, Netherlands.

[44] Rozek A, Powers JP, Friedrich CL, Hancock RE. 2003. Structure-based design of an indolicidin peptide analogue with increased protease stability. Biochemistry 42:14130–14138. doi:10.1021/bi035643g. 14640680

[45] Mahlapuu M, Håkansson J, Ringstad L, Björn C. 2016. Antimicrobial peptides: an emerging category of therapeutic agents. Front Cell Infect Microbiol 6:194. doi:10.3389/fcimb.2016.00194. 28083516 PMC5186781

[46] Corzo G, Escoubas P, Villegas E, Barnham KJ, He W, Norton RS, Nakajima T. 2001. Characterization of unique amphipathic antimicrobial peptides from venom of the scorpion *Pandinus imperator*. Biochem J 359:35–45. doi:10.1042/0264-6021:3590035. 11563967 PMC1222119

[47] Galanth C, Abbassi F, Lequin O, Ayala-Sanmartin J, Ladram A, Nicolas P, Amiche M. 2009. Mechanism of antibacterial action of dermaseptin B2: interplay between helix-hinge-helix structure and membrane curvature strain. Biochemistry 48:313–327. doi:10.1021/bi802025a. 19113844

[48] Rawson KM, Lacey M, Strong PN, Miller K. 2018. Improvements in bacterial selectivity following amino acid substitutions in Smp24, a venom derived AMP, poster 604. Microbe 2018. American Society for Microbiology, Washington, DC.

[49] Chen CH, Lu TK. 2020. Development and challenges of antimicrobial peptides for therapeutic applications. Antibiotics 9:24. doi:10.3390/antibiotics9010024. PMC7168295 31941022

[50] Clark GC, Casewell NR, Elliott CT, Harvey AL, Jamieson AG, Strong PN, Turner AD. 2019. Friends or foes? Emerging impacts of biological toxins. Trends Biochem Sci 44:365–379. doi:10.1016/j.tibs.2018.12.004. 30651181

[51] Nan YH, Shin SY. 2011. Effect of disulphide bond position on salt resistance and LPS-neutralizing activity of α-helical homo-dimeric model antimicrobial peptides. BMB Rep 44:747–752. doi:10.5483/BMBRep.2011.44.11.747. 22118542

[52] Shagaghi N, Clayton AH, Aguilar MI, Lee TH, Palombo EA, Bhave M. 2020. Effects of rationally designed physico-chemical variants of the peptide PuroA on biocidal activity towards bacterial and mammalian cells. Int J Mol Sci 21:8624. doi:10.3390/ijms21228624. PMC7696940 33207639

[53] Zhou K, Zhou L, Lim QE, Zou R, Stephanopoulos G, Too H-P. 2011. Novel reference genes for quantifying transcriptional responses of Escherichia coli to protein overexpression by quantitative PCR. BMC Mol Biol 12:18. doi:10.1186/1471-2199-12-18. 21513543 PMC3110127

[54] Andrews JM. 2001. Determination of minimum inhibitory concentrations. J Antimicrob Chemother 48:5–16. doi:10.1093/jac/48.suppl_1.5. 11420333

