# Supplementary figures and images for: Scorpion Venom Antimicrobial Peptides Induce Siderophore Biosynthesis and Oxidative Stress Responses in Escherichia coli
Source: mSphere. 2021 May 12;6(3):e00267-21. doi: 10.1128/mSphere.00267-21 (PMC8125054; doi:10.1128/mSphere.00267-21)

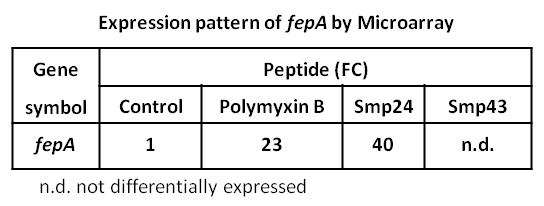


***fepA* relative expression level**

**B**


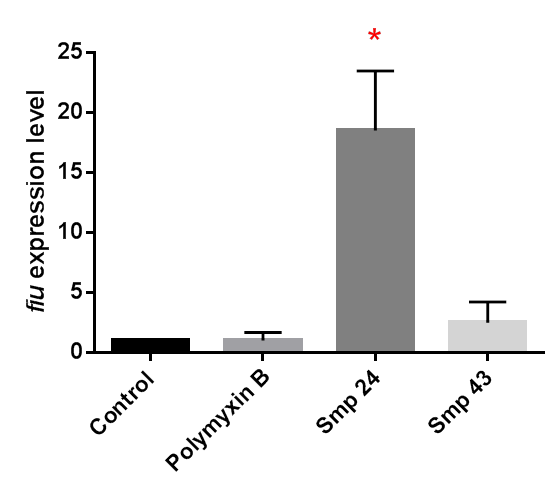


**AB**

***fiu* relative expression level**

**
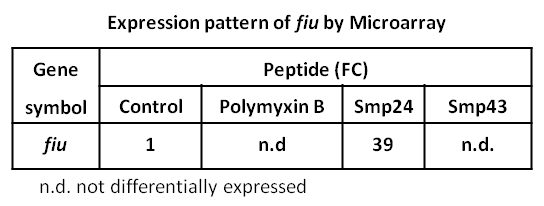
**

Figure S2.

Supplement: FIG S2 [file mSphere.00267-21-sf002.docx]

**
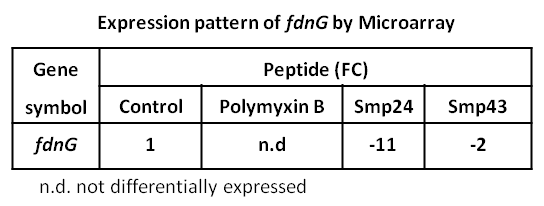
**

**B**


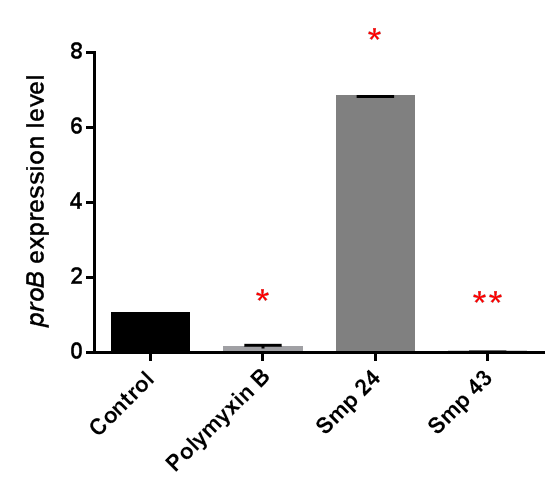


**A**


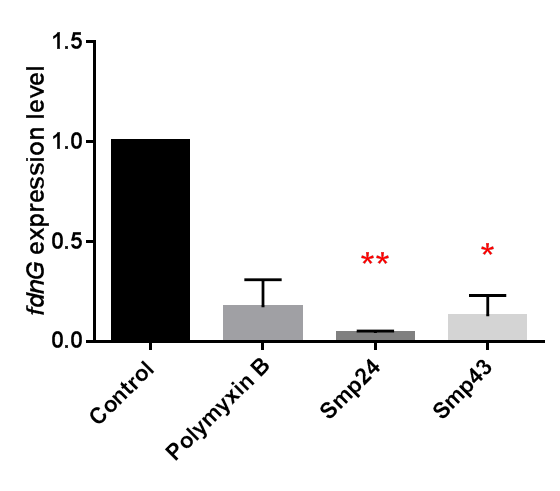


***fdnG* relative expression level**

***proB* relative expression level**

**
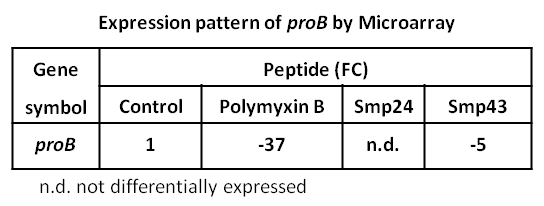
**

Figure S3.

Supplement: FIG S3 [file mSphere.00267-21-sf003.docx]
